# Timed up and go test and long-term survival in older adults after oncologic surgery

**DOI:** 10.1186/s12877-022-03585-4

**Published:** 2022-12-05

**Authors:** Sharon Hendriks, Monique G. Huisman, Frederico Ghignone, Antonio Vigano, Nicola de Liguori Carino, Eriberto Farinella, Roberto Girocchi, Riccardo A. Audisio, Barbara van Munster, Geertruida H. de Bock, Barbara L. van Leeuwen

**Affiliations:** 1 Department of Surgery, University of Groningen, University Medical Center Groningen, 9713 GZ Groningen, The Netherlands; 2 Department of Colorectal and General Surgery, Ospedale per gli Infermi, Faenza, Italy; 3 McGill Nutrition and Performance Laboratory, McGill University, Montreal, Canada; 4 Manchester Royal Infirmary, Department of Hepato-Pancreato-Biliary Surgery, Central Manchester University Hospitals, Manchester, UK; 5 Department of General Surgery and Surgical Oncology, University of Perugia, Hospital of Terni, Terni, Italy; 6 Department of Surgery, Institute of Clinical Sciences, Sahlgrenska University Hospital, Goteborg, Sweden; 7 Department of Internal medicine, University Medical Center Groningen, Groningen, The Netherlands; 8 Department of Epidemiology, University of Groningen, University Medical Center Groningen, Groningen, The Netherlands

**Keywords:** Older adults, Timed up and go, Physical performance, Long-term survival, Oncologic surgery

## Abstract

**Background:**

Physical performance tests are a reflection of health in older adults. The Timed Up and Go test is an easy-to-administer tool measuring physical performance. In older adults undergoing oncologic surgery, an impaired TUG has been associated with higher rates of postoperative complications and increased short term mortality. The objective of this study is to investigate the association between physical performance and long term outcomes.

**Methods:**

Patients aged ≥65 years undergoing surgery for solid tumors in three prospective cohort studies, ‘PICNIC’, ‘PICNIC B-HAPPY’ and ‘PREOP’, were included. The TUG was administered 2 weeks before surgery, a score of ≥12 seconds was considered to be impaired. Primary endpoint was 5-year survival, secondary endpoint was 30-day major complications. Survival proportions were estimated using Kaplan-Meier curves. Cox- and logistic regression analysis were used for survival and complications respectively. Hazard ratios (aHRs) and Odds ratios (aOR) were adjusted for literature-based and clinically relevant variables, and 95% confidence intervals (95% CIs) were estimated using multivariable models.

**Results:**

In total, 528 patients were included into analysis. Mean age was 75 years (SD 5.98), in 123 (23.3%) patients, the TUG was impaired. Five-year survival proportions were 0.56 and 0.49 for patients with normal TUG and impaired TUG respectively. An impaired TUG was an independent predictor of increased 5-year mortality (aHR 1.43, 95% CI 1.02-2.02). The TUG was not a significant predictor of 30-day major complications (aOR 1.46, 95% CI 0.70-3.06).

**Conclusions:**

An impaired TUG is associated with increased 5-year mortality in older adults undergoing surgery for solid tumors. It requires further investigation whether an impaired TUG can be reversed and thus improve long-term outcomes.

**Trial registration:**

The PICNIC studies are registered in the Dutch Clinical Trial database at www.trialregister.nl: NL4219 (2010-07-22) and NL4441 (2014-06-01). The PREOP study was registered with the Dutch trial registry at www.trialregister.nl: NL1497 (2008-11-28) and in the United Kingdom register (Research Ethics Committee reference 10/H1008/59). https://www.hra.nhs.uk/planning-and-improving-research/application-summaries/research-summaries/?page=15&query=preop&date_from=&date_to=&research_type=&rec_opinion=&relevance=true.

**Supplementary Information:**

The online version contains supplementary material available at 10.1186/s12877-022-03585-4.

## Background

The global burden of cancer is rapidly increasing, the number of new cancer cases among older adults is expected to double by 2035 compared to 2012 [1]. Cancer is a disease of ageing, and solid types of cancer predominantly affect the aged [2, 3]. For many of these solid types of cancer, surgery remains the most efficient treatment [4]. However, the ability to withstand major stressors like surgery varies greatly in the geriatric population undergoing oncologic surgery. Whilst older adults considered fit for surgery might do as well as younger patients, vulnerable or frail patients are at an increased risk of adverse postoperative outcomes [5–7]. Predicting which patient is at risk for postoperative adverse outcomes remains difficult and thus the management of older adults with solid cancer is challenging.

To improve postoperative outcomes in this group of patients, multiple interventions have been developed: prehabilitation is intended to enhance the functional capacity prior to surgery, enabling to withstand stressful events like surgery [8]. Prehabilitation consists mainly of endurance and resistance exercises [9]. Previous studies suggest that preoperative exercise may have beneficial effects on length of hospital stay, functional recovery and postoperative complications [10–12]. However, data on functional capacity in older adults undergoing oncologic surgery are scarce.

Before studying the effects of exercise as part of prehabilitation on long term survival in older adults going for oncologic surgery, it is essential to be informed on baseline physical functioning and long-term survival in these patients. Physical performance and functioning appear to be a proxy for the health status in older adults [13]. Functional tests can integrate known and unrecognized disturbances in multiple organ systems, such as heart, lungs, circulatory and musculoskeletal systems [13, 14]. One of the tests used to objectify physical performance is the Timed Up & Go test. This is a time-saving, inexpensive and easy to administer screening tool for physical performance, integrating information on multiple domains, such as gait speed, balance and strength [15]. In older oncological patients, the TUG seems to be an interesting indicator of the ability to withstand stressors such as chemotherapy or oncologic surgery [13]. All preceding studies have investigated the TUG as a screening tool in older adults undergoing oncological surgery focusing on short-term outcomes, such as length of hospital stay and complication rate [16–19].

Conversely, the primary objective of this study was to investigate the association between the TUG as a measurement of physical performance and 5-year survival after oncologic surgery.

## Methods

### The PICNIC, PICNIC B-HAPPY and PREOP studies

The data for this study concerns the long-term follow-up of three prospective observational cohort studies, ‘PICNIC’ (Postoperative Cognitive dysfunction In elderly Cancer patients), ‘PICNIC B-HAPPY’ (Biomarkers and Handgrip Strength as Predictors of Postoperative Outcome in PICNIC) and ‘PREOP’ (Preoperative Risk Estimation for Onco-geriatric Patients). The PICNIC studies were conducted at the University Medical Center Groningen (UMCG, The Netherlands) and approved by the Medical Ethical Committee of the University Medical Centre Groningen. The prospective international multicenter ‘PREOP’ (Preoperative Risk Estimation for Onco-geriatric Patients) cohort was designed by the surgical task force of the International Society of Geriatric Oncology and conducted between September 2008 and October 2012. The PRE-OP study was originally approved by the National Research Ethics Service Committee North West – Greater Manchester Central and the Medical Ethical Committee from Leiden University Medical center. The PREOP study was coordinated by the University Medical Center Groningen. Centers of the PRE-OP study participating in this study were the Orsola Malphighi Hospital, Bologna, Italy, University Medical Center Groningen, Groningen, The Netherlands, The Highfield Hospital, Manchester, United Kingdom, *S. Maria* Hospital Perugia, Italy and the McGill University Health Centre in Montreal, Canada. All patients gave written informed consent in accord with the ethical standards of the local ethics committees. Data collection was conducted according to the revised version of the Declaration of Helsinki (October 2013, Brazil). Patients from the PICNIC, PICNIC B-HAPPY and PREOP cohorts were described earlier [17, 20–28].

All clinical data such as age, sex, BMI, tumor type, disease stage, comorbidities, and surgical characteristics were prospectively collected from the patient’s medical record. During hospital admission complications were recorded prospectively. To complete the 30-days morbidity registration, patients’ files were checked on the occurrence of complications. Survival data for patients in ‘PICNIC’ and ‘PICNIC B-HAPPY’ was gathered from the patients’ medical record in December 2020.

Within 2 weeks prior to the surgical procedure, the TUG was administered as part of a larger test battery in all three studies [17, 20]. The TUG assesses the time a patient needs to get up from a chair, walk 3 m, turn around, walk back, and sit down again [15]. This is measured in seconds with a handheld stopwatch by the local researcher who performed the TUG two times for each patient; the mean of these measurements was then calculated. In literature, cut-off scores for an impaired TUG in older patients vary between 10 to 20 seconds [13, 17, 19, 29]. Focusing on survival in older adults after cancer treatment for various types of cancer, Hamaker et al. used 12 seconds, Soubeyran et al. used 20 seconds [18, 30]. Given this wide range in literature findings and the distribution of the TUG values in this current study, a score of equal of more than 12 seconds on the TUG was considered to be impaired.

### Inclusion and exclusion criteria

In this follow-up study, consecutive patients aged 65 and older undergoing elective surgery for solid malignant tumors were included in these analyses. Patients were excluded from analysis if histological examination of the tumor revealed a benign tumor, if the TUG had not been performed preoperatively or if surgery was scheduled in less than 24 hours after inclusion. A preoperative physical assessment was not used as an inclusion criterium in any of the studies, but patients who were physically unable to participate in this study and tests, were not included.

### Definitions and data collection

Several variables were taken into account in the here presented analysis. Age was included, sex, BMI in < 25 kg/m^2^ and ≥ 25 kg/m^2^, tumor stage by diagnose in a stage, and number of comorbidities in a group < 2 and a group of ≥2 comorbidities. Length of anesthesia duration during the operation was collected per 30 minutes. BMI and comorbidities were dichotomized based on clinical cut-offs and previous findings in literature [22, 25, 31].

### End points

The primary endpoint of this study was 5-year survival. The secondary endpoint was the incidence of any major 30-day complication, according to the Clavien Dindo (CD) classification (CD grade ≥ 3) conform previous comparable studies [32]. Major complications included those requiring surgical, endoscopic or radiological intervention (CD grade 3), life-threatening problems requiring Intensive Care management (CD grade 4) and death of a patient (CD grade 5). This secondary endpoint was analyzed as a dichotomous variable: major versus no or minor 30-day complications.

### Statistical analyses

Descriptive data were reported as absolute numbers and as percentages for categorical data. For continuous data, distributions were analyzed and mean and standard deviation (SD) or median and interquartile range (IQR) were given where appropriate. Overall 5-year survival was analyzed by Cox regression analysis. Median follow-up time was calculated by the Kaplan-Meier estimate of potential follow-up method [33]. A Log-Rank test was used to compare follow-up time for between groups with normal and impaired TUG. Kaplan-Meier curves were used to estimate 1- and 5-year survival proportions. Cox regression was used to estimate hazard ratios (HRs) and 95% confidence intervals (95% CIs). To estimate an adjusted HR (aHR), a multivariable cox regression analysis was carried out. In this multivariable analysis, we adjusted for sex, age, comorbidities (< 2 vs 2 or more), tumor stage and anesthesia time as measurement for the complexity of surgery. These variables were chosen based on clinical knowledge and preceding studies [20, 22, 34–36]. The aHR was also adjusted for the difference in cohorts (PICNIC and PREOP). To evaluate the incremental value of the TUG to the model without the TUG, we used a chi-square test of overall difference between log likelihoods of models.

Logistic regression analysis was used to estimate odds ratio’s (OR) and 95% confidence intervals (95% CI) for major complications. To estimate an adjusted OR (aOR) for the TUG regarding major complications, the OR was adjusted for sex, comorbidities (< 2 vs 2 or more) and anesthesia time as measurement for complexity of surgery [34–36]. The aOR was also adjusted for the cohorts (PICNIC, PICNIC B-HAPPY and PREOP). To evaluate adding the incremental value of the TUG to the model without the TUG, we used a chi-square test of overall difference between log likelihoods of models.

## Results

### Patient characteristics

**Fig. 1:**
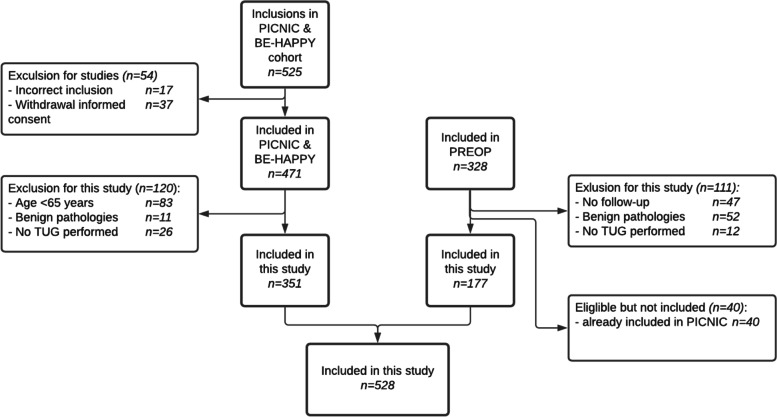
Flowchart of patients included in the study

Of the 525 patients originally included in the PICNIC & B-HAPPY cohorts and the 328 patients originally included in the PREOP cohort, 528 were over 65 years of age and older and were eligible for this study. In total, 83 patients were excluded based on age < 65 years, 63 patients turned out to have a benign diagnosis and in 38 patients TUG data were missing (Fig. 1). From the PREOP cohort, 177 patients (33.5%) were eligible for this study, from the PICNIC cohorts 351 patients (66.4%), of whom 109 (20.6%) were included in the B-HAPPY cohort.

Mean age was 75 years (SD 5.98) and 282 (53.4%) patients were female. Mean BMI was 26.7 Kg/m^2^ (SD 4.29) and median anesthesia time was 195 minutes (IQR 297). Mean TUG was 10.2 seconds (SD 5.57) and in 123 (23.3%) patients the TUG exceeded ≥12 sec (Table 1).

**Table 1:** Patient, disease and treatment-related characteristics of included patients (*n* = 528)

Variable		
Sex		
Female	282	(53.4)
Male	246	(46.6)
Age (years)	75	(6)
Body Mass Index (Kg/m^2^)		
≥ 25	327	(61.9)
Comorbidities present^a^		
Peripheral arterial diseases (including hypertension)	240	(45.5)
Cardiac diseases (incl. Atrial fibrillation)	134	(25.4)
Diabetes	94	(17.8)
Pulmonary diseases (asthma, COPD etc.)	71	(13.5)
Patients with 2 or more comorbidities	172	(32.6)
Tumor site		
Gastro-intestinal	245	(46.5)
Skin, soft tissue and lymph node	82	(15.6)
Breast	67	(12.7)
Gynecological	63	(12.0)
Hepatic, biliary and pancreatic	41	(7.8)
Para- and thyroid	16	(3.0)
Renal and bladder	8	(1.5)
Other	5	(0.9)
Tumor stage at inclusion		
I	192	(36.3)
II	84	(15.9)
IIII	136	(25.8)
IV	106	(20.1)
Anesthesia time (minutes)	195	[120-315]
Timed Up & Go test (seconds)		
≥ 12	123	(23.3)

^a^Comorbidities that occur most frequently are shown here

^b^for categories, N (%) is given, for continues variables, mean (SD) or median [1st-3th quartile] were given were appropriate

### Long-term survival

**Fig. 2:**
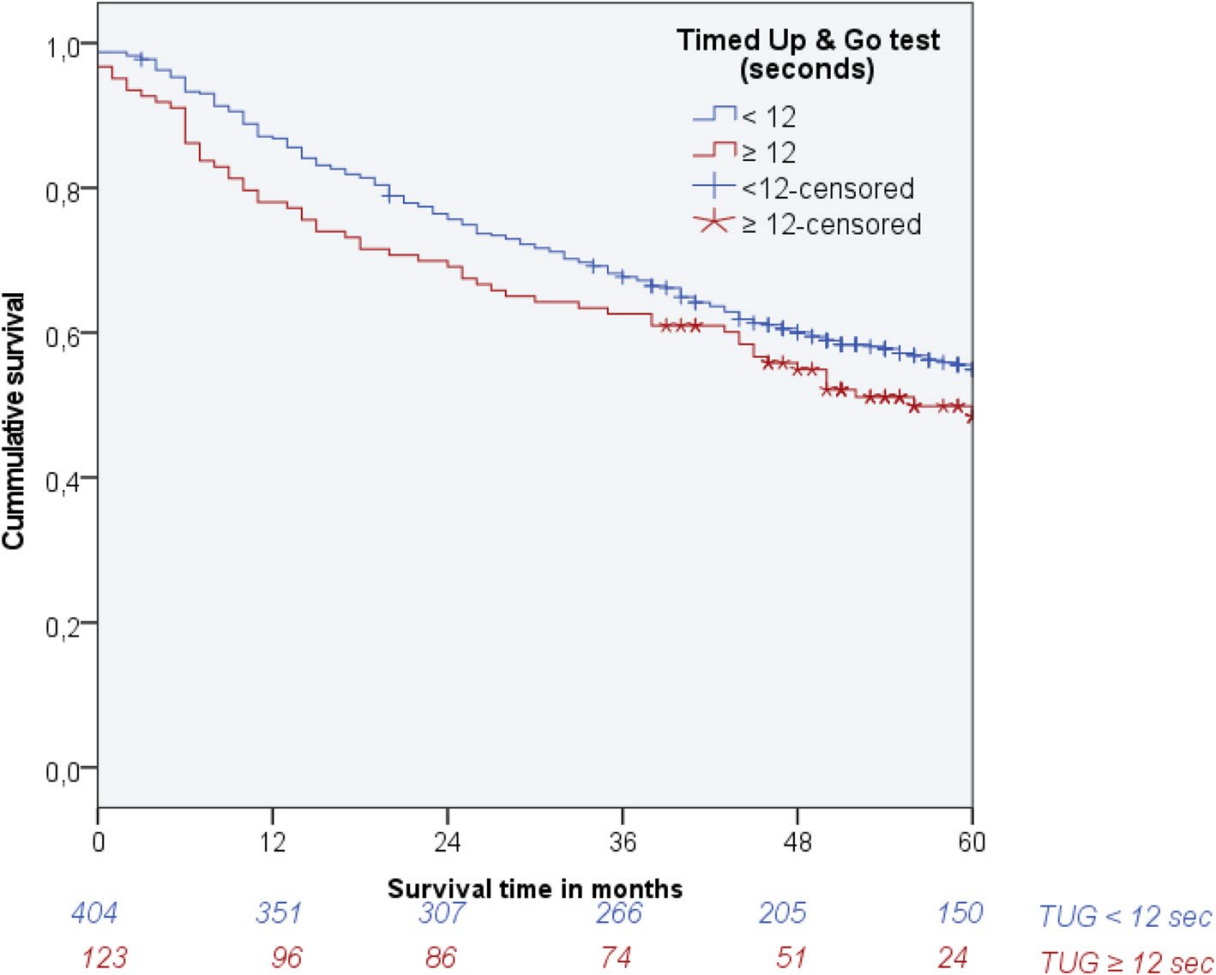
Overall survival of patients with a normal or a prolonged TUG (Number at risk per 12 months by patients with normal or a prolonged TUG)

The median follow-up time was 72 months (95% CI 66.1-77.9) and the overall postoperative survival proportion at 5 years was 0.54. The number of patients at risk at 5 years was 173. Figure 2 presents the Kaplan-Meier survival curve for the group of patients with a normal TUG and the group of patients with an impaired TUG, including numbers at risk per group. Survival proportions per time point in the group of patients with an impaired TUG were lower than survival proportions in the group of patients with normal TUG. Survival proportion at 5 years for normal TUG was 0.56, for impaired TUG 0.49 (Table 2; Log Rank test: P 0.056).

**Table 2:** Follow-up time and outcomes in the patients (1- and 5-years overall survival and major complications the first 30 days after surgery), overall and stratified for patients with a normal- or a prolonged TUG

	Follow-up time in months	Overall survival		Major 30-day complications
		1 year	5 years	
All (*n* = 528)	72.0 (66.1-77.9)	0.85	0.54	64 (12.1)
TUG < 12 (*n* = 405)	80.0 (71.8-88.2)	0.87	0.56	49 (12.2)
TUG ≥12 (*n* = 123)	62.0 (59.2-64.8)	0.79	0.49	15 (12.3)

Follow-up time is given as median (IQR), major complications as number (%). Overall survival proportions estimates are based on the Kaplan-Meier tables

**Fig. 3:**
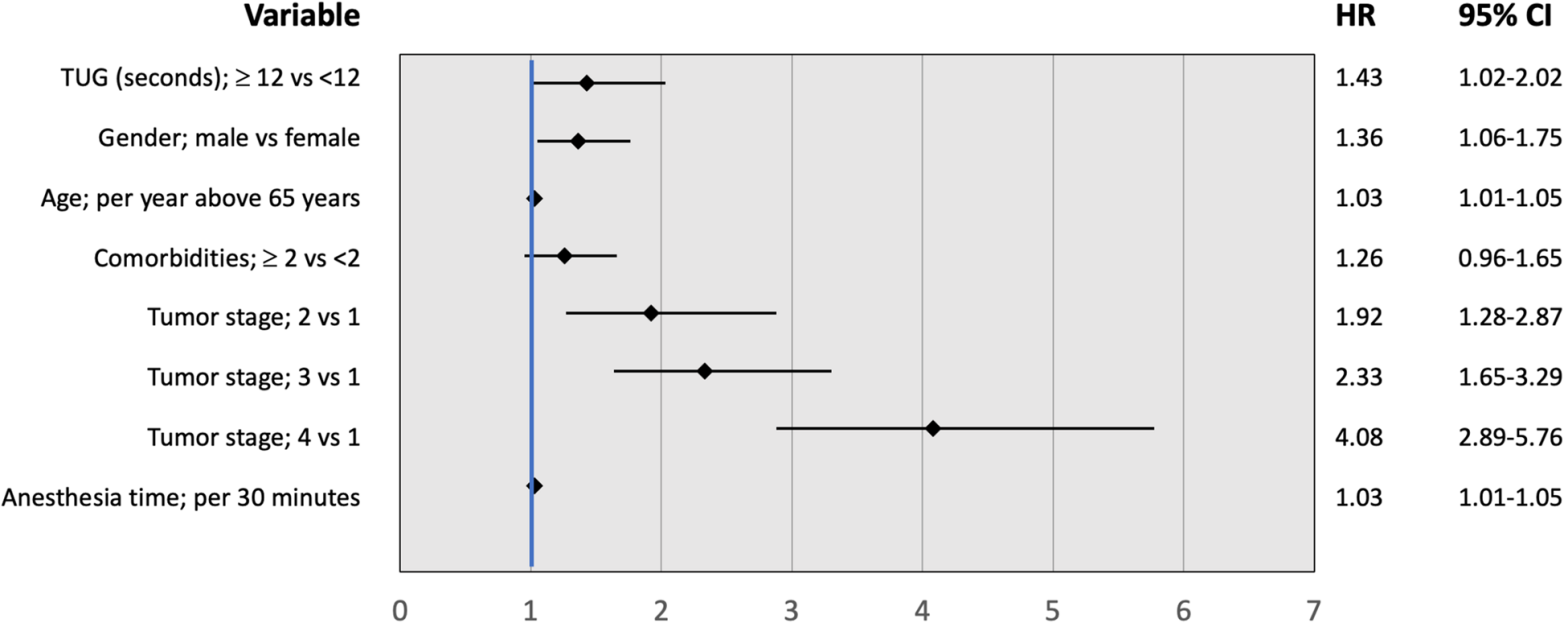
Baseline variables related to overall survival, multivariable analysis (Hazard ratios with 95% confidence intervals, also adjusted for cohorts)

See supplemental Table 1 for the results of the univariate analysis. In the multivariate model the following variables were included: sex, age, comorbidities, tumor stage, anesthesia time and TUG. An impaired TUG was a statistically significant predictor of mortality after adjustment for sex, comorbidities, tumor stage, anesthesia time (aHR 1.43, 95% CI 1.02-2.02, Fig. 3). When we compared a multivariable model with the TUG to a multivariable model without the TUG, it was seen that the TUG statistically significantly improved the model.

### 30-day complications

Major complications occurred in 64 patients (12.1%) within 30 days postoperatively. Major complications occurred in 49 patients (12.2%) with a normal TUG, and in 15 patients (12.3%) with an impaired TUG.

The multivariable model included the following variables: sex, comorbidities, anesthesia time and TUG. See supplemental Table 2 for the results of the univariate analysis.

**Fig. 4:**
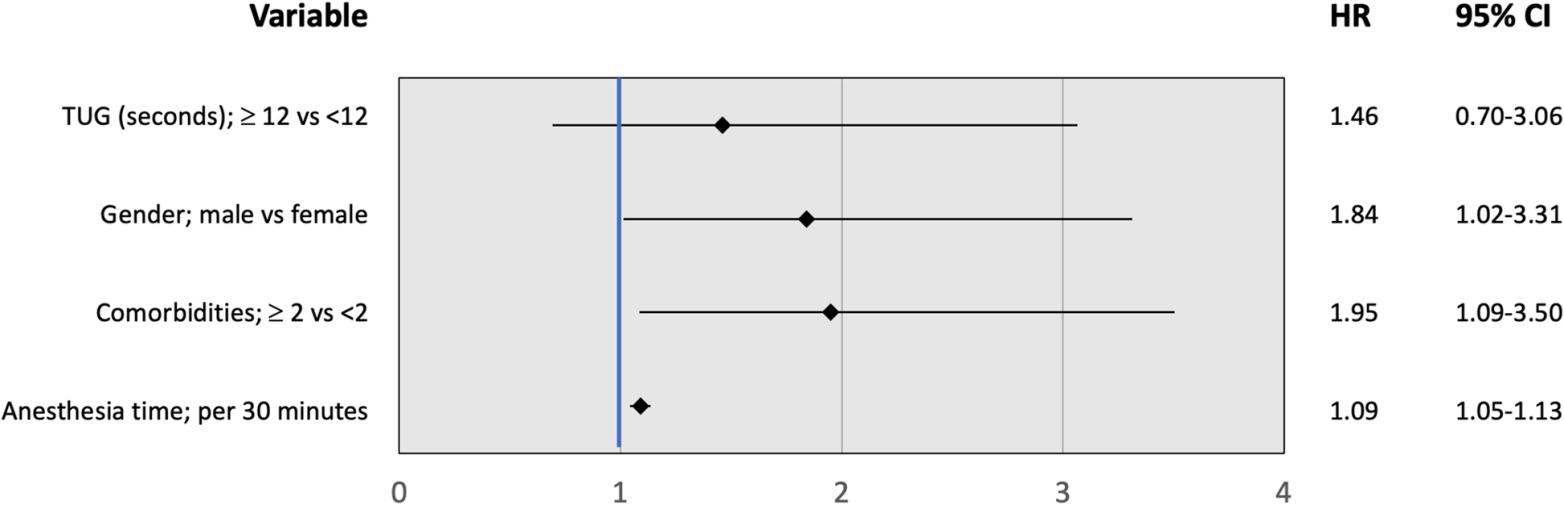
Baseline variables related to major complications, multivariable analysis (Odds ratios with 95% confidence intervals, also adjusted for cohorts)

In this model, the TUG was not a significant predictor of major complications within 30 days postoperatively (aOR 1.46, 95% CI 0.70-3.06, Fig. 4). The incremental value of the TUG to the literature-based model was therefore not calculated.

## Discussion

For 5-year mortality in older oncologic patients undergoing surgery, an impaired TUG was a predictor of increased mortality with an aHR of 1.43 (95% CI 1.02-2.02). No association was found between an impaired TUG and the occurrence of major complications 30 days postoperatively, aOR 1.46 (95% CI 0.70-3.06).

Similar to our findings, Ugolini et al. found a relation between impaired TUG and increased long term mortality. In this smaller group of patients with colorectal cancer (*n* = 46), an impaired TUG (> 20 sec) was a predictor of increased mortality with a HR of 3.51 in univariate analysis [37]. The higher HR in that study could be explained by the fact that the cut-off score for an impaired TUG was 8 seconds higher. Also, only univariate analysis was reported. The results we reported on 5-year mortality are in line with findings on 1-year mortality by Schmidt et al. in a group of 131 older oncologic patients after surgery. Schmidt et al. found a significantly higher 1-year mortality (OR 4.5, 95% CI 1.21-18.25) predicted by the combination of an impaired TUG (≥ 10 sec) with dependency in Activities of Daily Living (ADL, scores < 100) [19]. Robinson et al. also studied 1 year mortality in older patients undergoing colorectal and cardiac surgery with comparable findings. In the colorectal surgery group (*n* = 98), they found a 1-year mortality of 3% in the group of patients with a fast TUG (< 10 sec) compared to 31% in the group of patients with an impaired TUG (≥15 sec). For patients undergoing cardiac surgery (*n* = 174), 1-year mortality was 2% in the group of patients with a fast TUG compared to 12% for the group of patients with an impaired TUG [38]. The current study adds to these findings by studying a larger cohort with longer follow up times. Overall, TUG is a screening tool indicating an overall health in older patients prior to oncologic surgery, and an impaired TUG is able to predict postoperative mortality.

There are contradicting results in the relation between the TUG and 30-day complications. In this study, the occurrence of 30-day complications was used as an endpoint to be able to compare our results to the results of similar studies. Longer follow-up time could be more reflective of complications related to surgery. Being able to compare comparable our results to those of similar studies, aids the interpretation of results and implementation of the TUG in clinical practice. In this study, 64 patients (12.1%) experienced major complications, comparable to other studies investigating older oncologic surgical patients [39, 40]. An impaired TUG was not associated with the occurrence of major complications within 30-days postoperatively was found. In contrast to the findings in this study, Robinson et al. found that patients with an impaired TUG (≥ 15 sec) had significantly higher rates of complications postoperatively. In the colorectal group, 13% of the patients with a fast TUG (< 10 sec) had one or more complications, compared to 77% in the group with an impaired TUG (≥ 15 sec). In the cardiac surgery group, 11% of the patients with a fast TUG had one or more complications, compared to 52% in the group of patients with an impaired TUG [38]. Scholtz et al. studied a comparable group of 517 of patients aged ≥ 65 years. In this study, patients with an impaired TUG (≥20 sec) had a higher risk of overall complications (OR 2.59, 95% CI 1.05-6.39), but no association was found for major complications [39]. The incidence of major complications was comparable to our findings. In line with our findings, Martin et al. did not find an association between the TUG and complications after colorectal surgery, with a mean TUG of 9.0 sec (SD 2.9 sec) in the group without complications and a TUG of 9.9 sec (SD 2.9 sec) in the group with complications [41]. Where Robinson et al. and Scholtz et al. found an association between complication rate and TUG, we did not for major complications. Both studies used higher cut-off scores for an impaired TUG (≥ 15 sec and ≥20 sec respectively). In our study, the median TUG was 10.2 seconds, and 23.3% of the patients had an impaired TUG, where the cut-off was 12 seconds. This could mean that the cut-off of 12 seconds for an impaired TUG in our study was relatively low and a higher cut off is needed finding an association with complications. This could indicate that an impaired TUG can be used to predict total number of complications, but not for the prediction of major complications.

Some limitations and strengths need to be considered when interpreting the findings of this study. The strength of this study is that it is a large study including a large number of patients with various types of solid malignancies during a long follow up period. The patients that were included in this study might have had a better physical status and fewer co-morbidities than participants who refused to participate or for whom a surgical treatment did not seem to fit or for whom a surgical treatment did not seem fit [42]. Especially in the ‘PICNIC’ study, where one of the exclusion criteria was the presence of “any physical condition potentially impeding compliance with the study”.

The TUG can be seen as indicator of overall health in older adults and be helpful in the decision-making process. In this group of patients, other domains in addition to the physical one, need to be evaluated prior to surgical treatment [43]. Besides mortality, disability and lack of independence seem to impact patients with cancer more than the cancer prognosis per se [44]. In a survey by the Macmillan cancer support group in the UK for the older retired group of patients, continued independence was just as important as maintaining health [45]. In addition, Robinson and al found significantly higher rates of institutionalization (67%) in the group of patients with an impaired TUG (≥15 sec) compared to institutionalization rates (40%) of the group with a normal TUG (< 10 sec) [38]. Therefore, besides the effects of physical performance on outcomes such as complications and mortality, patient related-outcomes such as daily functioning and quality of life should be investigated in future studies too.

In addition to the TUG as a tool to aid the preoperative decision-making process, it might also indicate who can be in need of specialized rehabilitation postoperatively. Patients with an impaired TUG may have more difficulties restoring preoperative levels of physical functioning, because low fitness and/or low energy reserves make early mobilization more difficult. Also, malnourishment, sarcopenia and/or cachexia make these patients more vulnerable duo to a reduced capacity to increased demands for recovery of oncologic surgery [46]. Also, in older patients, early mobilization and enhanced recovery after surgery has been studied extensively and can be applied safely, with benefits such as reducing the occurrence of complications [47, 48]. A multidisciplinary approach, for example together with the experience of the geriatric department, is essential to achieve for example the encouragement to start early mobilizing and good nutrition and therefore recovery of surgery in this group of patients [47].

As an impaired TUG as measurement of physical performance is a predictor of increased mortality after surgery, it would be interesting to know whether an impaired TUG can be reversed and if improvement in survival is possible by interventions such as prehabilitation. Recent studies suggest that enhancing physical performance prior to surgery can accelerate post-operative mobilization and -recovery and reduce mortality. In older adult in major abdominal surgery, Barberan-Garcia et al. reported a significant reduction in overall complications in the intervention arm (20 of 62 versus 38 of 63 in the control arm). Patients in the intervention arm underwent a motivational interview, high-intensity endurance training and promotion of physical activity [49]. Boden et al. reported significantly lower rates of pulmonary complications in the intervention group (27 of 218 versus 58 of 214 in the control group, adjusted HR was 0.48, 95% CI 0.20-0.75) where intervention was physiotherapy education and breathing exercise training [50]. However, meta-analysis by Daniels et al. showed no significant difference in overall complications and pulmonary complications by prehabilitation [51]. The effect of prehabilitation on physical performance was studied by Bruns et al. by a meta-analysis [11]. They found that physical performance (walking distance, respiratory endurance) was improved in the prehabilitation (all trials included cardiopulmonary aerobic exercise) group, but did not find significant reduction of postoperative complications or length of hospital stay in the prehabilitation group [11]. The effects of prehabilitation on the TUG and long-term mortality in older adults going for oncologic surgery have not been extensively studied yet. In future studies, it would be interesting to see what types of prehabilitation can improve the TUG preoperatively and improve long-term survival especially in frail older oncological patients.

## Conclusions

An impaired TUG (> 12 sec) as measurement of physical performance is associated with increased long-term mortality in older adults undergoing oncologic surgery for various solid malignant tumors. No relation was found between an impaired TUG and major 30-day complications. The results of this study can be seen as baseline study on physical performance and long-term mortality. Future studies should focus on reversibility of an impaired TUG by prehabilitation.

## Supplementary Information

**Additional file 1.**

## Data Availability

The data that support the findings of this study are available on request from the corresponding author.

## Abbreviations

TUG: Timed Up and Go test
PICNIC: Postoperative Cognitive dysfunction In elderly Cancer patients
PICNIC B-HAPPY: Biomarkers and Handgrip Strength as Predictors of Postoperative Outcome in PICNIC
PREOP: Preoperative Risk Estimation for Onco-geriatric Patients
(a)HR: (adjusted) Hazard Ratio
CI: Confidence Interval
(a)OR: (adjusted) Odds Ratio
BMI: Body Mass Index
CD: Clavien Dindo classification
IQR: Inter Quartile Range
