# Supplementary material for: Timed up and go test and long-term survival in older adults after oncologic surgery
Source: BMC Geriatr. 2022 Dec 5;22:934. doi: 10.1186/s12877-022-03585-4 (PMC9720967; doi:10.1186/s12877-022-03585-4)
Supplement: Supplementary file 1 — Additional file 1. [file 12877_2022_3585_MOESM1_ESM.docx]

| **Supplementary table 1: Hazard ratio’s and 95% confidence intervals for univariate analysis for 5-year morbidity** | | | |
| --- | --- | --- | --- |
| **Variable** | **HR** | **CI 95%** | **P-Value** |
| TUG (seconds)   - <12 - ≥ 12 | reference  1.48 | 1.10-2.00 | 0.01 |
| Sex   - Female - Male | reference  1.48 | 1.17-1.89 | 0.001 |
| Age per year above 65 years | 1.03 | 1.01-1.05 | 0.002 |
| Comorbidities (number)   - <2 - ≥2 | reference  1.45 | 1.13-1.86 | 0.004 |
| Tumor stage   - Stage 0&1 - Stage 2 vs 1 - Stage 3 vs 1 - Stage 4 vs 1 | Reference  1.99  2.40  4.30 | 1.33-2.97  1.71-3.38  3.06-6.05 | <0.001  <0.001  <0.001  <0.001 |
| Anesthesia time per 30 minutes | 1.04 | 1.02-1.06 | <0.001 |

*All univariate analysis were adjusted for cohorts.*

| **Supplementary table 2: Odds ratio’s and 95% confidence intervals for univariate analysis for major complications.** | | | |
| --- | --- | --- | --- |
| **Variable** | **HR** | **CI 95%** | **P-Value** |
| Tug   - <12 - ≥12 | reference  1.30 | 0.66-2.55 | 0.45 |
| Sex   - Female - Male | reference  2.30 | 1.32-4.00 | 0.003 |
| Comorbidities (number)   - <2 - ≥2 | reference  2.19 | 1.27-3.77 | 0.005 |
| Anesthesia time per 30 minutes | 1.10 | 1.06-1.14 | <0.001 |

*All univariate analysis were adjusted for cohorts.*
